# Assessing the Efficacy and Acceptability of a Web-Based Intervention for Resilience Among College Students: Pilot Randomized Controlled Trial

**DOI:** 10.2196/20167

**Published:** 2020-11-11

**Authors:** Angel Enrique Roig, Olwyn Mooney, Alicia Salamanca-Sanabria, Chi Tak Lee, Simon Farrell, Derek Richards

**Affiliations:** 1 E-mental Health Research Group School of Psychology Trinity College Dublin, The University of Dublin Dublin Ireland; 2 Clinical Research & Innovation SilverCloud Health Dublin Ireland; 3 Future Health Technologies Programme, Campus for Research Excellence and Technological Enterprise, Singapore-ETH Centre Singapore Singapore

**Keywords:** web-based intervention, resilience, well-being, positive psychology, human support, automated support, college students, randomized controlled trial

## Abstract

**Background:**

College students are at elevated risk for developing mental health problems and face specific barriers around accessing evidence-based treatment. Web-based interventions that focus on mental health promotion and strengthening resilience represent one possible solution. Providing support to users has shown to reduce dropout in these interventions. Further research is needed to assess the efficacy and acceptability of these interventions and explore the viability of automating support.

**Objective:**

This study investigated the feasibility of a new web-based resilience program based on positive psychology, provided with human or automated support, in a sample of college students.

**Methods:**

A 3-armed closed pilot randomized controlled trial design was used. Participants were randomized to the intervention with human support (n=29), intervention with automated support (n=26), or waiting list (n=28) group. Primary outcomes were resilience and well-being, respectively measured by the Connor–Davidson Resilience Scale and Pemberton Happiness Index. Secondary outcomes included measures of depression and anxiety, self-esteem, and stress. Outcomes were self-assessed through online questionnaires. Intention-to-treat and per-protocol analyses were conducted.

**Results:**

All participants demonstrated significant improvements in resilience and related outcomes, including an unexpected improvement in the waiting list group. Within- and between-group effect sizes ranged from small to moderate and within-group effects were typically larger for the human than automated support group. A total of 36 participants began the program and completed 46.46% of it on average. Participants were generally satisfied with the program and found it easy to use.

**Conclusions:**

Findings support the feasibility of the intervention. Preliminary evidence for the equal benefit of human and automated support needs to be supported by further research with a larger sample. Results of this study will inform the development of a full-scale trial, from which stronger conclusions may be drawn.

**Trial Registration:**

International Standard Randomized Controlled Trial Number (ISRCTN) 11866034; http://www.isrctn.com/ISRCTN11866034

**International Registered Report Identifier (IRRID):**

RR2-10.1016/j.invent.2019.100254

## Introduction

The transition to university represents a period of increased academic and social pressure, financial burden, and change in lifestyle for students, placing them at increased risk for developing mental health problems [1]. The 12-month prevalence rate for mental disorders among college students is an estimated 20% yet only a small proportion of these students receive adequate treatment [2]. Given exposure to new sources of stress, the transitional nature of higher education settings affords a unique and timely opportunity to develop in students the skills needed to cope with new challenges [3]. In line with an increasing emphasis on promotion and prevention in mental health care [4], an approach that builds resilience against these stressors and prevents the development of mental disorders in the first instance may prove preferable to treatment following their onset [5]. Resilience may be understood as the personal assets (internal factors, eg, optimism) and environmental resources (external factors, eg, social support) that contribute to positive psychological adaption, despite exposure to adversity [6]. Resilience has been shown to buffer the effects of stress and burnout and protect against the development of depression, anxiety, and other common mental health problems [7,8].

Resilience interventions seek to promote resilience at an individual, group, or population level with the aim of preparing individuals for the occurrence of future life stressors [9]. This usually takes place through the enhancement of one or more resilience factors (assets and resources) [6]. However, the guiding theoretical framework and related techniques used in these interventions varies (eg, positive psychology, acceptance and commitment therapy, mindfulness, interpersonal therapy, and cognitive behavioral therapy), with no single accepted approach [9,10]. Given their inherent focus on promoting positive adaption and well-being, interventions based on positive psychology are highly compatible within the area of mental health promotion [11]. Several meta-analyses on positive psychology interventions have demonstrated significant improvements in well-being with small to moderate effect sizes [12,13]. For resilience interventions specifically, similar effect sizes have been observed in adults for resilience outcomes [9,14,15]. Encouragingly, initial research on resilience interventions among college students has demonstrated significant improvements in resilience and reductions in stress and symptoms of depression and anxiety [16-18].

An important consideration with the implementation of preventive interventions is ensuring that they can be accessed as widely as possible [11]. The internet is increasingly being used to deliver and improve the availability of interventions for mental health and well-being [19-22]. They may also prove particularly advantageous with students who are heavily immersed in the digital age [5]. Encouragingly, research investigating technology-delivered preventive interventions with college students has demonstrated small to moderate effect sizes for mental health outcomes [19,20] and preliminary support exists for the efficacy of web-based resilience interventions [23]. However, overall, research on web-based resilience interventions is scarce, particularly in youth samples, suggesting that further trials investigating their feasibility are necessary [14].

Despite the advantages of internet-delivery, one of the greatest limitations of web-based interventions are the high rates of dropout that can occur in these interventions [24]. Several meta-analyses show that supported web-based interventions yield lower rates of dropout and better clinical outcomes than unguided interventions [21,22,25,26]. Support may involve clarifying program content, monitoring progress, or motivating users either online or by phone or email [27]. Notably, the type of supporter, therapist or otherwise, has no bearing on effects [28]. This suggests that the greatest benefit resides in providing some form of contact, and opening the possibility of automating this contact [27]. Automated support is differentiated from human support in the related literature in that it is provided automatically through information and communication technologies (eg, automated reminders or feedback) [29]. While initial findings show that automated support is associated with slightly poorer rates of treatment efficacy and adherence compared to human support [28,30], continued improvements in developing high-quality automated support can lead to comparable outcomes, while reducing therapist time and implementation costs [27,29]. Preference for one type of intervention over another (eg, cognitive behavioral versus interpersonal therapy or individual versus group therapy) also has the potential to influence outcomes [31]. For example, increasing evidence shows significant improvements in clinical outcomes and higher rates of treatment adherence and satisfaction when participant preference for and allocation to an intervention group is matched [31,32].

Supported web-based resilience interventions are a promising, cost-effective approach for promoting resilience and preventing the onset of mental health problems. These interventions address issues relating to the accessibility of mental health care and may be of particular benefit to at-risk populations such as college students. This study sought to investigate the preliminary efficacy and acceptability of a new web-based intervention for resilience and well-being, based on positive psychology, in a sample of college students. The study also aimed to determine the effects of different types of support (human and automated) in the intervention on a range of outcomes including resilience, well-being, depressive and anxiety symptoms, self-esteem, and perceived stress.

## Methods

### Study Setting

The study was conducted in Trinity College Dublin (TCD) the University of Dublin, Ireland, in collaboration with the TCD Student Counselling Service and SilverCloud Health. TCD is a university for all of the major disciplines in the arts and humanities, and in business, law, engineering, science, and health sciences. The study was advertised to all registered students via email, posters, and social media. Students considering participating in the study were invited to visit an online platform through a URL where they received information about the study. Consent was obtained via digital signature. Recruitment started in February 2019 and the trial ran for 4 consecutive months, until June 2019 when data collection was completed.

### Research Design

A 3-armed, parallel-group, pilot randomized controlled trial design was used. Using an allocation ratio of 1:1:1, participants were randomized to the intervention with human support, intervention with automated support, or waiting list control group. The randomization schedule was generated by an individual independent of the study via sequentially numbered, opaque sealed envelopes using *Random Allocation Software* [33]. Randomization was performed in blocks of 12 with 3 groups. Given the nature of the trial, participants and researchers were not blinded to group allocation. The CONSORT-EHEALTH guidelines [34] (Multimedia Appendix 1) were followed and the study protocol has been published [35].

### Sample Size

Previous data on effect sizes for web-based resilience interventions in college students do not exist [5]. However, findings from 2 meta-analyses on resilience and positive psychology interventions demonstrated small effect sizes for resilience and psychological well-being, respectively [12,14]. A sample size of 25 per arm is recommended for pilot trials when effect sizes are expected to be small [36]. This calculation is based on a main trial designed with 90% power and 2-sided 5% signiﬁcance. Given a 3-armed pilot trial design and small anticipated effect size for resilience and well-being, a sample size of 75 (25 participants per arm) was determined.

### Eligibility Criteria

Inclusion criteria were being over the age of 18 and a registered student at TCD. Exclusion criteria were currently attending counselling or psychotherapy, having an organic mental health condition, or being at risk of suicide.

### Intervention

Space for Resilience is a 7-module program aimed at promoting resilience and well-being through the enhancement of several well-evidenced resilience factors [6]. The program was developed by SilverCloud Health in line with the principles of positive psychology [37] and incorporates cognitive behavioral elements including cognitive flexibility, optimism, challenging negative self-talk, behavioral activation, and active coping, alongside information on social support, lifestyle factors, and values. Modules are structured in an identical way and include introductory videos, quizzes, psychoeducational content, personal stories from other users, interactive activities, mindfulness exercises, homework suggestions, goal setting, and summaries. A description of module content is provided in Multimedia Appendix 2 [6,8,37-46] and a screenshot of the program is provided in Multimedia Appendix 3. The program was offered over an 8-week intervention period and was accessible 24/7. It was recommended that participants spend at least an hour a week on the program based on previous studies with the same platform [47,48].

### Support

#### Human

Participants in the human support group were assigned to a supporter from the TCD Student Counselling Service. Supporters were counsellors or trainee counsellors familiar with using the SilverCloud Health platform and received training in the *Space for Resilience* program. The role of the supporter was to monitor and support user progress through the program. On the supporter interface of the platform, an overview of each users’ level of engagement with the program is presented. This includes user responses on questionnaires, messages left by the user, module pages viewed, tools and activities used (including content shared by the user), and the number of times the user logged in to the platform. Using this information, supporters spent 10-15 minutes formulating individualized reviews for each participant. Reviews are asynchronous messages sent and received on the platform. Supporters received guidelines on how to support users. These guidelines advise that in every review, the supporter should (1) demonstrate empathy and care to the user, (2) demonstrate knowledge of the theory underlying the program, (3) acknowledge and affirm the user’s progress, (4) prompt and encourage further use of the program, (5) ask reflective questions, and (6) set homework. Participants received 4 reviews during the intervention period. An excerpt from a sample review is provided below:

Well done for logging in to the Space for Resilience programme again this week. I can see you completed the second module, Self, which supports you in identifying your values, passions, and what matters most to you in life. Did any questions come up for you during this module?

I noticed from one of the tools you filled in that building your social network is something you would like to focus on. The Connections module might be particularly helpful for this. It includes useful information on developing relationships and building communities as well as tips for improving communication skills like active listening and expressing gratitude.

Remember, applying the skills you lean in this module to your everyday life is like building up a muscle. You might not see the reward straight away but the more time you spend on it, the more your social network will grow and the stronger your communication skills will become.

#### Automated

Participants in the automated support group received generic, templated reviews which were automatically sent as messages on the platform. Automated reviews were designed to facilitate user progress through the program and were structured in the same way as reviews in the human support group (eg, users are encouraged to explore new content in the program). However, reviews in the automated support group were standardized as opposed to individualized. They were therefore not tailored to each user’s unique level of engagement with the program. Automated reviews were predeveloped by highly experienced clinicians with in-depth knowledge of providing support for web-based interventions. Participants received 4 reviews during the intervention period. An excerpt from an automated review is provided below:

Have you been finding the programme useful so far? No matter how much time you have spent exploring the programme since your last review, we wanted to remind you that even a small effort can make a big difference.

You can complete the modules in whatever order suits you best. Over the next two weeks, we suggest that you work through one or two more of the five domains of resilience modules: purpose, self, connections, body, or mind.

Remember that this programme is designed to help you, but it is up to you to make the changes. Do what you can, one step at a time. 

### Measures

All outcomes were self-assessed through online questionnaires. See Table 1 for measure administration timeline.

**Table 1:** Measure administration timeline.

Measure	Measurement point	
	Baseline	Postintervention
Sociodemographic and Clinical History Questionnaire	X	
Connor–Davidson Resilience Scale (CD-RISC)	X	X
Pemberton Happiness Index (PHI)	X	X
Brief Resilience Scale (BRS)	X	X
Patient Health Questionnaire—4 Items (PHQ-4)	X	X
Rosenberg Self-Esteem Scale (RSES)	X	X
Perceived Stress Scale—4 Items (PSS-4)	X	X
Satisfaction With Treatment (SAT)^a^		X

^a^Only the active intervention groups completed the SAT.

#### Screening Measure: The Sociodemographic and Clinical History Questionnaire

The Sociodemographic and Clinical History Questionnaire [49] collects sociodemographic information including age, gender, education level, and computer literacy; and clinical information including current engagement with counselling or psychotherapy, drug and alcohol use, diagnosis of an organic mental health condition, and suicide risk. While group assignment was random, this questionnaire included an item asking participants if they would prefer to receive human or automated support and why.

#### Primary Outcome Measures

##### Connor–Davidson Resilience Scale

The Connor–Davidson Resilience Scale (CD-RISC) [50] is a 25-item self-report measure of resilience or ability to cope with stress. The CD-RISC has shown good concurrent validity and internal consistency (α=.89) with college students [51].

##### Pemberton Happiness Index

The Pemberton Happiness Index (PHI) [52] is a 21-item self-report integrative measure of well-being. Of these items, 11 relate to remembered well-being (ie, general, hedonic, eudaimonic, and social well-being) and 10 relate to experienced well-being (ie, positive and negative events that happened the previous day). The PHI has demonstrated good convergent and incremental validity and strong internal consistency (α>.89) [52].

#### Secondary Outcome Measures

##### Brief Resilience Scale

The Brief Resilience Scale (BRS) [53] is a 6-item self-report measure assessing resilience or ability to bounce back or recover from stress. The BRS has shown strong convergent validity and good internal consistency (α>.80) with college students [53].

##### Patient Health Questionnaire—4 Items

The Patient Health Questionnaire—4 Items (PHQ-4) [54] is a brief self-report measure of depression and anxiety. The PHQ-4 has demonstrated good construct and criterion validity and internal consistency (α=.81) with college students [55].

##### Rosenberg Self-Esteem Scale

The Rosenberg Self-Esteem Scale (RSES) [56] is a 10-item self-report measure of global self-esteem. The RSES has shown good construct validity, internal consistency (α=.87), and test–retest reliability (r=.84) with college students [57].

##### Perceived Stress Scale—4 Items

The Perceived Stress Scale—4 Items (PSS-4) [58] is a brief self-report measure of the extent to which recent life events are considered stressful. The PSS-4 has demonstrated acceptable criterion validity and internal consistency (α=.72) [58].

#### Other Measures

##### Platform Usage Metrics

Usage refers to the degree to which participants were exposed to the intervention [59]. Related data for active intervention groups were collected automatically on the online platform. This included number of logins to the program, length of time spent using the program, number of tools and activities used, and percentage of program content viewed. A session was defined as any instance where a participant logged in to the platform. Number of sessions was therefore determined by the total number of participant logins.

##### Satisfaction With Treatment

The Satisfaction With Treatment (SAT) [60] is an 8-item self-report measure of attitudes toward the web-based intervention. It also includes 2 open-ended questions asking participants what they most and least liked about the intervention.

#### Procedure

After providing informed consent, participants completed baseline measures. Participants meeting eligibility criteria were randomized to the human support, automated support, or waiting list group and were informed of group assignment immediately. Active intervention groups were given immediate access to the program. The waiting list group was given access to the program after an 8-week waiting period. To minimize dropout, participants received a phone call from a member of the research team (CTL and SF) approximately 1 week following randomization to remind them of group assignment and research procedures. After the 8-week period, participants received an email asking them to complete postintervention measures. Participants were informed of institutional affiliations during the informed consent procedure and were not reimbursed for their participation in the trial.

#### Data Analysis

Data were analyzed using SPSS software (version 24) [61]. Recruitment and retention rates were examined using descriptive statistics and a Pearson chi-square test. Sociodemographic information and baseline data were examined using descriptive statistics, Pearson chi-square tests, and one-way analysis of variance (ANOVAs). Reliability checks using Cronbach α were conducted on outcome measures.

Intention-to-treat (ITT) and per-protocol analyses were conducted on primary and secondary outcomes measures. Per-protocol analysis considered all participants who completed baseline and postintervention outcome measures and, in the case of active intervention groups, accessed the program at least once. For ITT analysis, missing data were calculated using the expectation-maximization algorithm, a maximum likelihood method used in similar trials [62]. Preliminary efficacy was evaluated using mixed factorial ANOVAs. Within- and between-group effect sizes (Cohen *d*) and 95% confidence intervals were calculated for each group. Effect sizes of 0.2 were considered small, 0.5 were considered medium, and 0.8 were considered large [63]. The use of ANOVAs represents a revision to study protocol which outlined the use of linear mixed models in the analysis plan [35]. However, diagnostic tests on the data revealed inadequate power and model fit to sufficiently address the research questions under investigation. Mixed factorial ANOVAs were therefore deemed more suitable. There was a modest departure from the assumption of homogeneity of variance; however, the *F*-test has shown to be robust against moderate departures and variance heterogeneity is frequently observed in real-world data [64].

Usage data were analyzed using descriptive statistics, Pearson chi-square tests, and unpaired *t* tests. Data from the SAT were analyzed using descriptive statistics, unpaired *t* tests, and descriptive and interpretive analysis [65]. Descriptive and interpretive analysis is an integrative approach to analyzing qualitative data that aims to identify and analyze patterns in the data by delineating meaning units and organizing them into categories. Between-group effect sizes (Cohen *d*) and 95% confidence intervals were also calculated for usage and SAT data.

To explore the effects of intervention preference and allocation, exploratory subgroup analyses were conducted. Participants in the active intervention groups were divided into 2 groups: those who were allocated to their preferred intervention group and those who were not. Pearson chi-square tests, ANOVAs, and unpaired *t* tests were used to examine differences in outcomes, engagement and usage, and satisfaction with the intervention.

#### Ethical Considerations

The study received full ethical approval from the TCD School of Psychology Research Ethics Committee on January 29, 2019 (approval ID: SPREC112018-12). Ethical considerations are fully outlined in the study protocol [35].

#### Data Sharing

Data will be made available upon request to the corresponding author.

## Results

### Recruitment and Retention

**Figure 1:**
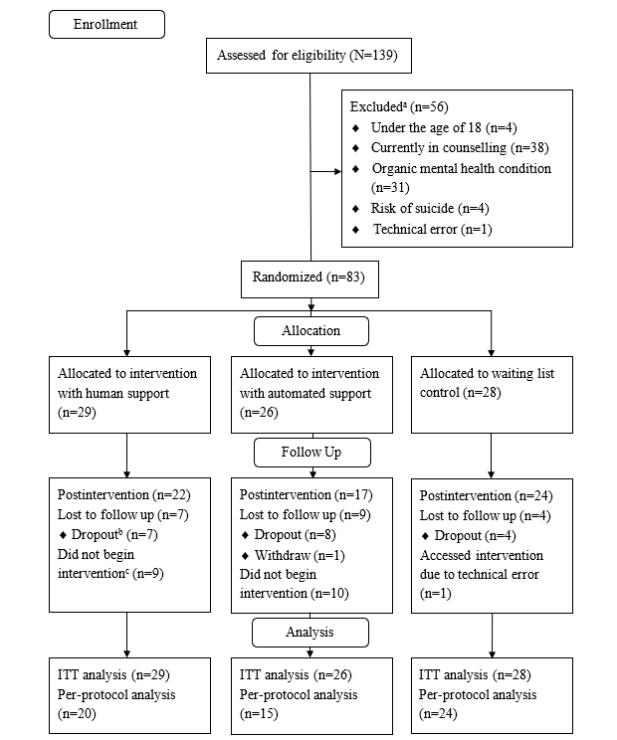
CONSORT flow diagram. ITT: intention to treat. aSome participants met more than 1 reason for exclusion and are categorized as such. bRefers to participants that did not complete postintervention measures. cRefers to participants who did not start the intervention.

Out of the estimated 17,000 students invited, 139 (0.82%) students signed up to participate and were assessed for eligibility. A total of 83 (59.7%) participants met eligibility criteria and were included in the trial. Of these, 63 (76%) completed postintervention measures. The dropout rate was therefore 24% (20/83). A Pearson chi-square test revealed no differences between groups in terms of completion of outcome measures at postintervention. Participant flow through the trial is presented in Figure 1.

### Baseline Characteristics

The median age of participants was 26 years (IQR 11). In terms of computer literacy, most participants were either confident or very confident in using computers and the internet (73/82, 89%). Baseline characteristics of the sample are provided in Table 2. Pearson chi-square tests and one-way ANOVAs demonstrated no significant differences between groups in terms of sociodemographic variables or scores on baseline measures. Reliability checks demonstrated satisfactory internal consistency for all outcome measures (α>.70).

**Table 2:** Baseline characteristics of study sample.

Characteristic		Human support (N=29), n (%)		Automated support (N=26), n (%)		Waiting list (N=28), n (%)		Total (N=83), n (%)	
**Gender (N=81)**									
	Male		6 (21)		4 (15)		4 (15)		14 (17)
	Female		22 (79)		22 (85)		23 (85)		67 (83)
**Education level (N=82)**									
	Undergraduate		6 (21)		4 (15)		0 (0)		10 (12)
	Postgraduate		9 (31)		7 (27)		13 (48)		29 (35)
	Other studies		14 (48)		15 (58)		14 (52)		43 (52)
**Computer literacy (N=82)**									
	Very confident		14 (50)		15 (58)		17 (61)		46 (56)
	Confident		11 (39)		9 (35)		7 (25)		27 (33)
	Average		2 (7)		2 (8)		3 (11)		7 (9)
	Mildly confident		1 (4)		0 (0)		1 (4)		2 (2)
	Not confident		0 (0)		0 (0)		0 (0)		0 (0)
**Intervention preference**									
	Human support		22 (76)		17 (65)		16 (57)		55 (66)
	Automated support		7 (24)		9 (35)		12 (43)		28 (34)

### Preliminary Efficacy

Descriptive statistics, within- and between-group effect sizes, and confidence intervals for ITT and per-protocol analyses are presented in Table 3.

**Table 3:** Descriptive statistics, effect sizes, and confidence intervals for intention-to-treat and per-protocol analysesa.

Measure (Construct)			Baseline, mean (SD)	Postintervention, mean (SD)	Within-group effect size, *d* (95% CI)	Between-group effect size, *d* (95% CI)
**Intention to treat** **(N=83)**						
	**CD-RISC^a^** **(Resilience)**					
		HS^b^	60.28 (14.02)	66.63 (11.63)	0.44 (–0.08 to –0.97)	HS vs AS: 0.02 (–0.51 to 0.55)
		AS^c^	62.58 (13.50)	66.43 (11.02)	0.50 (–0.05 to 1.06)	AS vs WL: –0.14 (–0.68 to 0.39)
		WL^d^	61.46 (12.63)	68.17 (12.95)	0.60 (0.07 to 1.14)	HS vs WL: –0.13 (–0.65 to 0.40)
	**PHI^e^** **(Well-being)**					
		HS	6.02 (1.45)	6.79 (0.99)	0.46 (–0.07 to –0.98)	HS vs AS: 0.38 (–0.15 to 0.91)
		AS	6.10 (1.59)	6.41 (1.01)	0.25 (–0.30 to 0.79)	AS vs WL: –0.46 (–1.00 to 0.08)
		WL	6.68 (1.33)	6.91 (1.15)	0.18 (–0.34 to 0.71)	HS vs WL: –0.11 (–0.63 to 0.41)
	**BRS^f^** **(Resilience)**					
		HS	3.00 (0.70)	3.24 (0.54)	0.57 (–0.05 to 1.10)	HS vs AS: –0.02 (–0.55 to 0.51)
		AS	3.15 (0.69)	3.25 (0.63)	0.24 (–0.31 to 0.78)	AS vs WL: 0.09 (–0.44 to 0.63)
		WL	2.98 (0.72)	3.18 (0.84)	0.31 (–0.22 to 0.84)	HS vs WL: 0.09 (–0.43 to 0.61)
	**PHQ-4^g^** **(Depression/Anxiety)**					
		HS	4.72 (3.14)	3.93 (2.58)	–0.23 (–0.75 to 0.28)	HS vs AS: –0.02 (–0.54 to 0.51)
		AS	4.31 (3.22)	3.89 (2.65)	–0.19 (–0.73 to 0.36)	AS vs WL: –0.38 (–0.92 to 0.16)
		WL	3.61 (2.44)	2.95 (2.25)	–0.38 (–0.90 to 0.15)	HS vs WL: –0.40 (–0.93 to 0.12)
	**RSES^h^** **(Self-esteem)**					
		HS	26.79 (5.92)	28.51 (5.27)	0.53 (0.01 to 1.05)	HS vs AS: 0.04 (–0.49 to 0.57)
		AS	27.27 (5.02)	28.34 (3.79)	0.34 (–0.21 to 0.88)	AS vs WL: –0.55 (–1.10 to –0.01)
		WL	29.71 (5.46)	30.89 (5.27)	0.37 (–0.16 to 0.90)	HS vs WL: –0.45 (–0.98 to 0.07)
	**PSS-4^i^** **(Perceived stress)**					
		HS	8.41 (3.52)	7.36 (2.46)	–0.30 (–0.81 to 0.22)	HS vs AS: –0.08 (–0.61 to 0.45)
		AS	7.31 (2.40)	7.17 (2.18)	–0.07 (–0.61 to 0.48)	AS vs WL: –0.34 (–0.87 to 0.20)
		WL	6.93 (2.89)	6.27 (3.06)	–0.42 (–0.95 to 0.11)	HS vs WL: –0.39 (–0.92 to 0.13)
**Per protocol (N=59)**						
	**CD-RISC (Resilience)**					
		HS	59.40 (15.40)	63.90 (10.98)	0.37 (–0.25 to 1.00)	HS vs AS: –0.11 (–0.78 to 0.56)
		AS	63.80 (13.19)	65.20 (12.21)	0.20 (–0.52 to 0.92)	AS vs WL: –0.24 (–0.88 to 0.41)
		WL	61.38 (12.39)	68.29 (13.66)	0.61 (0.03 to 1.19)	HS vs WL: –0.35 (–0.95 to 0.25)
	**PHI (Well-being)**					
		HS	6.02 (1.42)	6.63 (0.96)	0.45 (–0.18 to 1.08)	HS vs AS: 0.23 (–0.45 to 0.90)
		AS	6.34 (0.96)	6.39 (1.18)	0.07 (–0.65 to 0.79)	AS vs WL: –0.42 (–1.07 to 0.23)
		WL	6.64 (1.33)	6.90 (1.23)	0.20 (–0.37 to 0.77)	HS vs WL: –0.24 (–0.84 to 0.36)
	**BRS (Resilience)**					
		HS	2.95 (0.76)	3.18 (0.63)	0.50 (–0.13 to 1.13)	HS vs AS: 0.02 (–0.65 to 0.69)
		AS	3.13 (0.78)	3.17 (0.66)	0.09 (–0.63 to 0.80)	AS vs WL: 0.04 (–0.61 to 0.68)
		WL	2.92 (0.72)	3.14 (0.89)	0.33 (–0.24 to 0.90)	HS vs WL: 0.05 (–0.54 to 0.65)
	**PHQ-4 (Depression/Anxiety)**					
		HS	4.90 (3.19)	3.70 (2.27)	–0.39 (–1.01 to 0.24)	HS vs AS: –0.01 (–0.68 to 0.66)
		AS	3.80 (2.91)	3.67 (2.82)	–0.06 (–0.77 to 0.66)	AS vs WL: –0.28 (–0.93 to 0.37)
		WL	3.71 (2.51)	2.96 (2.40)	–0.41 (–0.99 to 0.16)	HS vs WL: –0.32 (–0.91 to 0.28)
	**RSES (Self-esteem)**					
		HS	26.55 (5.98)	28.35 (5.69)	0.49 (–0.14 to 1.12)	HS vs AS: –0.04 (–0.71 to 0.63)
		AS	28.07 (3.95)	28.53 (3.09)	0.11 (–0.60 to 0.83)	AS vs WL: –0.48 (–1.13 to 0.17)
		WL	29.46 (5.24)	30.75 (5.35)	0.40 (–0.17 to 0.97)	HS vs WL: –0.44 (–1.04 to 0.17)
	**PSS-4 (Perceived stress)**					
		HS	9.00 (3.42)	7.65 (2.60)	–0.33 (–0.95 to 0.30)	HS vs AS: –0.15 (–0.82 to 0.53)
		AS	6.87 (2.33)	7.27 (2.63)	0.17 (–0.54 to 0.89)	AS vs WL: –0.31 (–0.96 to 0.34)
		WL	7.13 (2.69)	6.33 (3.20)	–0.56 (–1.14 to 0.02)	HS vs WL: –0.45 (–1.05 to 0.15)

^a^CD-RISC: Connor–Davidson Resilience Scale.

^b^HS: human support.

^c^AS: automated support.

^d^WL: waiting list.

^e^PHI: Pemberton Happiness Index.

^f^BRS: Brief Resilience Scale.

^g^PHQ-4: Patient Health Questionnaire—4-items.

^h^RSES: Rosenberg Self-Esteem Scale.

^i^PSS-4: Perceived Stress Scale—4-items.

#### ITT Analysis

Mixed factorial ANOVAs demonstrated main effects of time for resilience (CD-RISC; *F*_1,80_=21.56, *P*<.001), well-being (PHI; *F*_1,80_=9.40, *P*=.003), resilience (BRS; *F*_1,80_=10.08, *P*=.002), depressive and anxiety symptoms (PHQ-4; *F*_1,80_=5.96, *P*=.02), self-esteem (RSES; *F*_1,80_= 15.18, *P*<.001), and perceived stress (PSS-4; *F*_1,80_=5.48, *P*=.02). No interaction effects or main effects of group were observed for any outcome measure. For main effects of time, mean scores show an increase in resilience, well-being, and self-esteem and decrease in depressive and anxiety symptoms and perceived stress for all participants.

#### Per-Protocol Analysis

Mixed factorial ANOVAs demonstrated main effects of time for resilience (CD-RISC; *F*_1,56_=9.16, *P*=.004), well-being (PHI; *F*_1,56_=4.20, *P*=.045), resilience (BRS; *F*_1,56_=4.26, *P*=.04), depressive and anxiety symptoms (PHQ-4; *F*_1,56_=5.34, *P*=.03), and self-esteem (RSES; *F*_1,56_=6.51, *P*=.01). No main effect of time was observed for perceived stress (PSS-4). No interaction effects or main effects of group were observed for any outcome measure. For main effects of time, mean scores demonstrate an increase in resilience, well-being, and self-esteem and decrease in depressive and anxiety symptoms for all participants.

### Acceptability

#### Engagement and Usage

A total of 36/55 (65%) participants in the active intervention groups started the program. A Pearson chi-square test revealed no significant difference between groups in terms of whether or not participants started the intervention. The mean number of sessions was 8.50 (SD 3.65) and average session length was 20.38 minutes (SD 8.95). On average, participants spent a total of 171.55 minutes (SD 101.36) on the program and completed 46.46% (SD 27.80) of the program. Computers were the preferred device for accessing the program (64.30% of total use), followed by mobiles (33.52% of total use) and tablets (2.18% of total use). Independent *t* tests demonstrated no significant differences in engagement and usage between active intervention groups. Descriptive statistics, between-group effect sizes, and confidence intervals for program engagement and usage are presented in Table 4, with effect sizes generally favoring the human support group.

**Table 4:** Descriptive statistics for program engagement and usage.

Variable	Human support, (N=20), mean (SD)	Automated support, (N=16), mean (SD)	Total, (N=36), mean (SD)	Between-group effect size, *d* (95% CI)
Number of sessions	9.40 (2.93)	7.38 (4.22)	8.50 (3.65)	0.57 (–0.10 to 1.24)
Length of program use per session (in minutes)	20.14 (1.89)	20.68 (2.45)	20.38 (8.95)	–0.25 (–0.91 to 0.41)
Total length of program use (in minutes)	197.15 (110.01)	139.56 (81.72)	171.55 (101.36)	0.58 (–0.09 to 1.26)
Number of tools used	8.40 (3.02)	6.50 (3.86)	7.56 (3.50)	0.56 (–0.11 to 1.23)
% program content viewed	48.95 (24.42)	43.35 (32.08)	46.46 (27.80)	0.20 (–0.46 to 0.86)
% computer use	56.94 (37.25)	73.50 (34.27)	64.30 (36.42)	–0.46 (–1.13 to 0.21)
% mobile use	40.91 (38.24)	24.29 (32.32)	33.52 (36.22)	0.47 (–0.20 to 1.13)
% tablet use	2.16 (6.74)	2.21 (8.83)	2.18 (7.62)	–0.01 (–0.66 to 0.65)

#### Satisfaction With the Intervention

A total of 34/55 (62%) participants in the active intervention groups started the program and completed the SAT. Independent *t* tests demonstrated no significant differences between active intervention groups in terms of scores on the SAT. Descriptive statistics, between-group effect sizes, and confidence intervals for the SAT are displayed in Table 5, with effect sizes nearly all in favor of the human support group. Both groups liked the flexibility, user-friendliness, and positive psychology approach of the program. The human support group identified liking anonymity and supporter feedback, with one participant reporting that simply “knowing there was support” [participant #17, male] was helpful. Both groups disliked the lack of face-to-face interaction. Participants also reported that the program did not fully meet their individual needs and wants. One participant noted that it “was quite vague at times” [participant #7, female] while another reported that it “felt too prescriptive in how life should be” [participant #35, female]. The human support group disliked the infrequent timing of reviews, reporting that they felt discontinuous. One participant in the automated support group reported that receiving human support may have encouraged greater use of the program. Several participants noted time restrictions and lacking motivation as barriers to program completion.

**Table 5:** Descriptive statistics for the SAT^a^.

Item	Human support, (N=19), mean (SD)	Automated support, (N=15), mean (SD)	Total, (N=34), mean (SD)	Between-group effect size, *d* (95% CI)
I was happy to use the computer to access treatment^b^	3.95 (0.85)	3.67 (0.90)	3.82 (0.87)	0.32 (–0.36 to 1.00)
I found the online treatment easy to use	4.16 (0.96)	4.00 (0.66)	4.09 (0.83)	0.19 (–0.49 to 0.87)
I felt the treatment received will have a long-lasting effect	3.26 (0.93)	3.33 (0.82)	3.29 (0.87)	–0.08 (–0.76 to 0.60)
I would recommend the online treatment to other users	3.82 (0.77)	3.40 (0.83)	3.65 (0.81)	0.53 (–0.16 to 1.22)
Please rate how helpful you found the online treatment program^c^	3.05 (0.62)	2.73 (0.70)	2.91 (0.67)	0.49 (–0.20 to 1.17)
How likely is it that you would recommend this treatment program to a friend or colleague?^d^	6.95 (2.53)	6.07 (2.66)	6.56 (2.58)	0.34 (–0.34 to 1.02)

^a^SAT: Satisfaction With Treatment.

^b^Score range=1-5 for items 1-4.

^c^Score range=1-4.

^d^Score range=1-10.

### Intervention Preference and Allocation

With regard to intervention preference, 66% of participants opted for human support (55/83). Main reasons for selecting human support included the belief that human contact cannot be replaced and perceptions that it would be more personalized and beneficial than automated support. Prominent reasons for opting for automated support included a want for greater privacy and an interest in the user experience of receiving automated support.

In terms of primary and secondary outcomes, mixed factorial ANOVAs demonstrated no significant difference between participants who were or were not assigned their preferred intervention, from baseline to post-intervention, for ITT (n=55) or per-protocol (n=35) analyses. However, a Pearson chi-square test (n=55) revealed that participants who were allocated their preferred intervention were significantly more likely to complete postintervention measures than those who were not (χ^2^_1_=5.8, *P*=.02).

Regarding engagement and usage, independent *t* tests (n=36) demonstrated a significant difference between participants who started the intervention in terms of length of program use (t_33.96_=3.45, *P*=.002) and number of logins (t_34_=2.15, *P*=.04). Participants who were allocated their preferred intervention tended to spend more time on the program (n=23; mean 205.42 [SD 106.13]) and log in more frequently (n=23; mean 9.43 [SD 3.06]) than participants who were not, who on average spent less time on the program (n=13; mean 111.64 [SD 56.82]), and logged in less frequently (n=13; mean 6.85 [SD 4.14]). Participants did not differ significantly on any other engagement and usage or satisfaction (n=34) variable.

Additional *post hoc* analyses revealed that participants who elected for and received human support spent significantly longer time on the program (n=16; mean 213.72 [SD 111.70]) than participants who elected for human support and received automated support (n=9; mean 103.10 [SD 44.40]; t_21.44_=3.50, *P*=.002). Similarly participants who elected for and received automated support spend significantly longer on the program (n=7; mean 186.44 [SD 97.52]) than participants who elected for automated support and received human support (n=9; mean 103.10 [SD 44.40]; t_14_=2.93, *P*=.04).

## Discussion

### Principal Findings

This pilot study investigated the preliminary efficacy and acceptability of a web-based intervention for resilience, provided with human or automated support, in a sample of college students. All participants demonstrated significant improvements in resilience, well-being, and self-esteem and reductions in symptoms of depression and anxiety, and perceived stress, thereby confirming the beneficial effects of the web-based resilience intervention. With regard to the role of support, the results are preliminary in nature, but results show overall equivalence of outcomes between human and automated support.

Effect sizes were generally moderate for resilience outcomes and small for well-being outcomes, in line with existing research on resilience and positive psychology interventions [9,12-18]. Similarly, for secondary outcomes of self-esteem, depression and anxiety symptoms, and perceived stress, effects ranged from small to moderate. Notably, effects for resilience tended to be larger on the BRS (which measures a resilient outcome) in the human support group and larger on the CD-RISC (which measures the assets and resources that lead to a resilient outcome) in the automated support group [66]. It is possible that the personalized element of human support facilitated the application of skills targeted by the intervention to participants’ specific life circumstances, increasing the likelihood of a resilient outcome. Comparably, while the automated support group likely developed these skills, they perhaps lacked the tailored support conducive to applying them, limiting the opportunity for a resilient outcome. However, these results are preliminary and a larger-scale trial is needed to confirm the direction of the findings.

The general equivalence of outcomes across the 2 active intervention arms is in contrast with research demonstrating more favorable outcomes when human support is provided [28,30]. However, this does compare to some preliminary evidence of comparable outcomes between human and automated support [27,29]. This may have been due to a greater sense of agency in the automated support group as participants were not dependent on a therapist and the quality of automated support [27,29]. Nonetheless, effect sizes tended to be larger for the human support group. This may have been due to the personalization of feedback in this group. Therefore, the addition of persuasive technology features such as tailoring or personalization to automated support may bring it up to par with human support in terms of effect [27].

Even more interesting was our finding that observed effects were likely impacted by user preference, demonstrating that those who opted for the human or automated supported intervention and received that had higher engagement. While intervention preference and allocation had no effect on intervention outcomes or satisfaction, participants who received their preferred intervention did use the program more and were more likely to complete postintervention measures. These findings support research showing higher levels of treatment adherence and retention when preference and allocation are matched [31,32]. This may point to the clinical utility of a shared model of decision making when more than 1 intervention option is available [31].

While our findings are, for the most part, in line with existing research, a nonsignificant difference between active intervention groups and controls is something that we did not expect. It is important to note that the effects observed in the active intervention groups cannot be attributed to the intervention with certainty, given significant improvements and comparable effect sizes in the waiting list group. This amelioration may have been due to a self-selection bias in this study whereby students who were more motivated to change signed up to participate [67]. Accordingly, anticipation effects, that is, changes in outcome due to expectation of future change, may have been present in the waiting list group [68]. As there was no follow-up, it cannot be determined if these effects dissipated over time or if effects in the active intervention groups were sustained. Lastly, given that the study was a pilot, sample size was small. Because of greater variability in participant responses, it is likely that the sample was not large enough to differentiate between groups [69].

An initial recruitment rate of less than 1% may be partially attributed to the fact that participants were not reimbursed for participating in the study. However, retention rates in this study were high resulting in a dropout rate of only 24%. This is impressive relative to other web-based intervention research which has demonstrated dropout rates of up to 83% [24]. In terms of program usage, 65% (36/55) of participants in the active intervention groups started the program and completed 46.46% of it on average. It is therefore possible that participants did not receive the full benefit of the intervention in this study, underestimating its true efficacy and further contributing to the nonsignificant difference between intervention and control groups. Potential reasons for low levels of engagement or dropout or both include the lack of time students had to spend on the program and insufficient support, which may have influenced motivation to use the intervention. Both of these were noted in the satisfaction data collected. Given its significant effect on usage, it is also viable that dropout rates were impacted by user preference and allocation to intervention groups.

Overall, participants were satisfied with the intervention, found it helpful and easy to use, and would recommend it to others. Participant satisfaction with the intervention did not vary based on the type of support provided, further indicating the equivalence of human and automated support. In line with previous research on web-based interventions [70], participants liked the flexibility and accessibility of the program and disliked not having enough time to complete it. As the human support group disliked the infrequent timing and discontinuation of reviews, it raises the question as to the role of support in such interventions; perhaps as has been suggested previously, there may be different routes to treatment success that is dependent on user characteristics and type of support required [71]. Knowledge regarding the amount of support necessary in preventive web-based interventions is currently unclear [17]. The decision to provide fortnightly reviews in this study was based on the fact that participants were not drawn from a clinical population and were deemed capable of completing the program with minimal support. However, in order to sufficiently motivate and support user engagement, it is possible that the same degree of support is necessary as in remedial programs, where support is typically implemented on a weekly basis [21,72].

### Limitations

The main limitation of the study was the small sample size. This resulted in greater heterogeneity in the data, leading to an unforeseen change to study protocol in terms of data analysis. However, it should be acknowledged that as the study is a pilot, establishing the true efficacy of the intervention was not the primary goal. Second, as data on time spent on the program per week were not collected, it was not possible to determine if participants adhered to the recommended dose of usage [47,48]. Further, reasons for participants not signing up to the intervention and dropout were not collected, limiting related insights around recruitment and attrition. As there was no follow-up assessment, the long-term effects of the intervention could not be gauged. Besides, the study did not examine the occurrence of adverse events following the intervention; change in resilience was based on self-report scales. Therefore, it cannot be determined whether or not participants had the opportunity to apply the skills acquired through the intervention by the time of postintervention assessment. Additionally, the sample included postgraduate students who may not be representative of a high-risk student population given that they are likely to be more well-adjusted than undergraduate students [73].

### Implications and Future Research

To the authors’ best knowledge, this is the first study to explore the role of human and automated support in a resilience intervention in a college sample. Primarily, the results of this pilot will inform the development and implementation of a full-scale trial. It is possible that more reviews should be provided to groups to increase engagement, with final reviews preparing the human support group for the discontinuation of support. As telephone calls and emails to participants from the research team constituted reminders about research procedures, their omission is not anticipated to affect outcomes or usage in routine application. Preliminary evidence for the equivalence of human and automated support must be replicated before related conclusions are drawn. Future research should further consider the effects of participant preference for support and the role of personalization in automated support, establish recommendations around intervention dose, and include follow-up assessment(s). Applications may then be considered, including the widespread implementation of the intervention at a universal level and for at-risk populations.

### Conclusion

Web-based interventions aimed at promoting resilience demonstrate an important protective function in mitigating the effects of stress. They have the potential to reduce the occurrence of mental health problems in those who are at heightened risk and experience difficulties around accessing adequate treatment. Beyond prevention, an emphasis on resilience reflects a larger shift in focus away from pathology and toward psychological well-being and human strengths. These interventions therefore play an important role in the area of mental health promotion in terms of increasing not only the emphasis placed on successful versus stressful life events, but also their prevalence.

## Appendix

Multimedia Appendix 1 CONSORT-EHEALTH checklist.

Multimedia Appendix 2 Space for Resilience: Description of module content.

Multimedia Appendix 3 Space for Resilience: Screenshot of the intervention.

## Abbreviations

ANOVA: analysis of variance
BRS: Brief Resilience Scale
CD-RISC: Connor–Davidson Resilience Scale
PHI: Pemberton Happiness Index
PHQ-4: Patient Health Questionnaire—4 Items
PSS-4: Perceived Stress Scale—4 Items
RSES: Rosenberg Self-Esteem Scale
SAT: Satisfaction With Treatment
