# Supplementary material for: Assessing the Efficacy and Acceptability of a Web-Based Intervention for Resilience Among College Students: Pilot Randomized Controlled Trial
Source: JMIR Form Res. 2020 Nov 11;4(11):e20167. doi: 10.2196/20167 (PMC7688384; doi:10.2196/20167)
Supplement: Multimedia Appendix 2 [file formative_v4i11e20167_app2.docx]

| Module name | Brief description |
| --- | --- |
| Building resilience | This opening module introduces the concept of resilience [6] and allows the user to analyse their current levels of resilience and set goals for the programme. The practice of mindfulness [39] and its relevance for resilience is also introduced. |
| Purpose | This module focuses on purpose, meaning, and values [38,40]. The user is encouraged to identify their values, what matters most to them in life, and their passions, and find ways to incorporate these into the key life roles they undertake. |
| Self | This module focuses on self-esteem and self-compassion. The user is encouraged to identify their strengths and align them to their values and passions [38]. Users are also invited to challenge their negative self-talk and replace it with more compassionate statements [41]. |
| Connections | This module supports users in reflecting on their social networks and improving their relationships and communities. Information about communication styles is provided and the user is given tips for improving their communication skills [42,43]. |
| Body | This module focuses on creating a healthy lifestyle [44] by developing positive habits for sleep, diet, and exercise. Behavioural activation techniques [45] are provided and the user can track their daily lifestyle choices and observe how they impact on their mood. |
| Mind | This module focuses on thoughts and offers balanced optimism [40,43] and gratitude [46] as alternatives to negative or distorted thinking [47]. |
| Moving forward | This final module looks at active coping methods [8] for dealing with problems and prepares the user for coming to the end of the programme. Users have the opportunity to review their progress since starting the programme and set goals for the future. |
