# Supplementary figures and images for: Assessing the Efficacy and Acceptability of a Web-Based Intervention for Resilience Among College Students: Pilot Randomized Controlled Trial
Source: JMIR Form Res. 2020 Nov 11;4(11):e20167. doi: 10.2196/20167 (PMC7688384; doi:10.2196/20167)

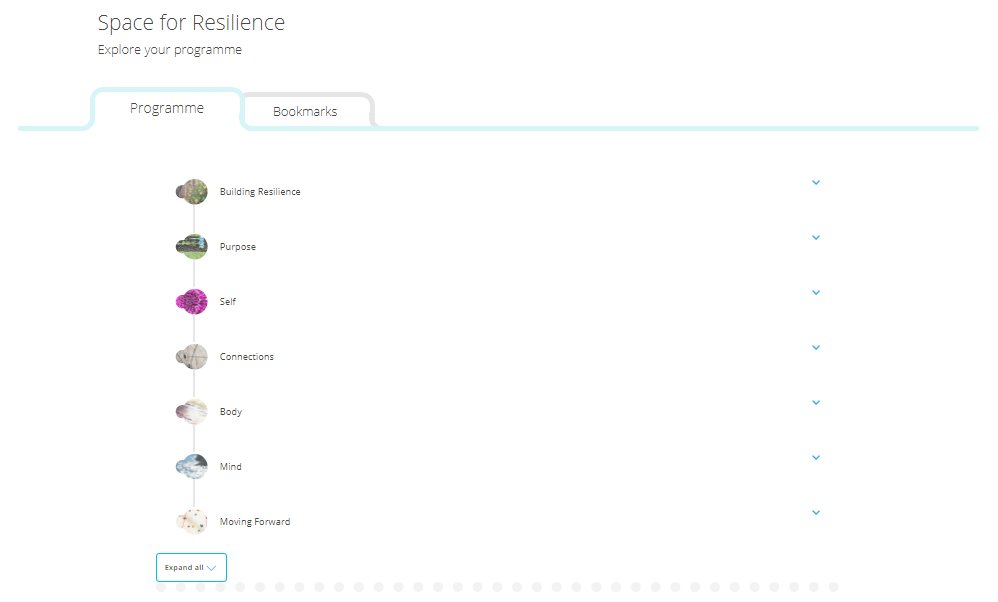

Supplement: Multimedia Appendix 3 [file formative_v4i11e20167_app3.png]
